# Effects of Filament Extrusion, 3D Printing and Hot-Pressing on Electrical and Tensile Properties of Poly(Lactic) Acid Composites Filled with Carbon Nanotubes and Graphene

**DOI:** 10.3390/nano10010035

**Published:** 2019-12-21

**Authors:** Giovanni Spinelli, Rumiana Kotsilkova, Evgeni Ivanov, Ivanka Petrova-Doycheva, Dzhihan Menseidov, Vladimir Georgiev, Rosa Di Maio, Clara Silvestre

**Affiliations:** 1 Department of Information and Electrical Engineering and Applied Mathematics, University of Salerno, Via Giovanni Paolo II, 84084 Fisciano (SA), Italy; 2 Institute of Mechanics, Bulgarian Academy of Sciences, Acad. G. Bonchev Str. Block 4, 1113 Sofia, Bulgaria; kotsilkova@yahoo.com (R.K.); ivanov_evgeni@yahoo.com (E.I.); ivanka.petrova01@gmail.com (I.P.-D.); cihan.menseidov@gmail.com (D.M.); 3 Research and Development of Nanomaterials and Nanotechnologies (NanoTech Lab Ltd.), Acad. G. Bonchev Str. Block 4, 1113 Sofia, Bulgaria; vladofe@gmail.com; 4 Institute of Polymers, Composites and Biopolymers, CNR, Via Campi Flegrei 34 A. Olivetti, 80078 Pozzuoli (NA), Italy; rosadimaio1988@libero.it (R.D.M.); clarasilvestre@yahoo.it (C.S.)

**Keywords:** MWCNT, GNP, hybrid composites, filament, 3D printing (FDM), TEM, DSC, electrical conductivity, tensile test

## Abstract

In this study, the effects of three processing stages: filament extrusion, 3D printing (FDM), and hot-pressing are investigated on electrical conductivity and tensile mechanical properties of poly(lactic) acid (PLA) composites filled with 6 wt.% of multiwall carbon nanotubes(MWCNTs), graphene nanoplatelets (GNPs), and combined fillers. The filaments show several decades’ higher electrical conductivity and 50–150% higher values of tensile characteristics, compared to the 3D printed and the hot-pressed samples due to the preferential orientation of nanoparticles during filament extrusion. Similar tensile properties and slightly higher electrical conductivity are found for the hot-pressed compared to the 3D printed samples, due to the reduction of interparticle distances, and consequently, the reduced tunneling resistances in the percolated network by hot pressing. Three structural types are observed in nanocomposite filaments depending on the distribution and interactions of fillers, such as segregated network, homogeneous network, and aggregated structure. The type of structural organization of MWCNTs, GNPs, and combined fillers in the matrix polymer is found determinant for the electrical and tensile properties. The crystallinity of the 3D printed samples is higher compared to the filament and hot-pressed samples, but this structural feature has a slight effect on the electrical and tensile properties. The results help in understanding the influence of processing on the properties of the final products based on PLA composites.

## 1. Introduction

In the last years, a growing interest has been found in the research of graphene and carbon nanotube reinforced polymer nanocomposites with superior properties for a variety of applications. So far, little is known about the effectiveness of the carbon-based polymer nanocomposites as a novel material for additive manufacturing (3D printing) [1]. In our previous studies on the poly(lactic) acid-based nanocomposite materials filled with graphene and carbon nanotubes for 3D printing application, variety of factors are reported to govern the final nanocomposite properties, such as types and combinations of fillers, processing conditions, state of dispersion, intrinsic interactions, nucleation effect of fillers on polymer crystallization, printing parameters, etc. [2,3,4]. Due to the complexity of those factors, a variety of contradicting properties may be obtained for the nanocomposite filament materials based on poly(lactic) acid (PLA). In line with structure-property relations, it was found that above a critical “percolation” threshold of nanofiller content, mechanical properties of nanocomposites are significantly improved, and electrical conductivity appears if conducting nanoparticles, such as graphene and carbon nanotubes are used [2,4,5]. It is well known that not only the filer concentration is critical for the properties of polymer nanocomposites, but also the distribution of filler in the matrix polymer is of significant importance. Recently, researchers found that the construction of a segregated structure by incorporating a small amount of highly conductive nanoparticles, such as carbon nanotubes and graphene in a polymer matrix, could fabricate composites with improved electrical conductivity at low percolation threshold over traditional randomly distributed composites [6]. Such segregated nanocomposites are usually prepared either by dispersion of filler in polymer blends or by the wrapping of polymer powder with nanoplatelets, such as carbon nanotubes and graphene followed by compressive molding [6]. Shi et al. [7] adopted a local enrichment strategy for the preparation of highly conductive PLA-carbon nanotube composites with segregated structure by extrusion.

The poly(lactic) acid (PLA) gained much attention in additive manufacturing by fused deposition modeling (FDM), because of its easy printability, relatively good mechanical properties, and biodegradability [1]. However, a weak point for the PLA-based filaments is that their mechanical properties depend strongly on the processing conditions, which influence the PLA crystallinity [8]. Researchers reported that carbon nanotubes (CNTs) and graphene nanoplatelets (GNPs) may enhance the nucleation rate of polylactide; thus, they can tune specific physical and mechanical properties by providing nucleation agents to initiate crystallization [8,9]. It is observed that the crystallinity decreases at higher cooling rates and higher GNP content, due to the slowly crystallizing PLA polymer and the GNPs aggregation [10]. A comparative crystallization study on PLA composites filled with CNTs and GNPs found that both nanofillers act as heterogeneous nucleation agents, however, the induction ability of CNTs is stronger than that of GNPs, due to the multiple orientations of PLA lamellae on the large two-dimensional flat surface of GNPs, which suppressed the crystal growth [11]. The carbon filler loading may affect the melting behavior and properties of PLA significantly, producing higher crystallinity and enhanced Young modulus, impact strength, flexural modulus, and stiffness [12]. Moreover, systematic effects on the electrical conductivity of carbon nanotube filled polymers were observed as a function of annealing temperature and time, and this phenomenon was related to the reorientation of nanotubes due to enhanced polymer mobility, that was named “dynamic percolation”, as opposed to traditional statistical percolation [13]. In our previous study [14], annealing was found efficient for improving mechanical, thermal and electrical properties of the aged PLA-based composite filaments with 12 wt.% carbon nanotubes and graphene nanoplatelets; however, the annealing temperature has to be tuned accordingly to the type of carbon nanofiller and the target properties.

Generally, the development of technologies for mass production of carbon-based controllable materials and products remains challenging, as the behavior, physical properties, and other functions of composites depend strongly on their microstructure obtained during processing. These phenomena must be studied systematically in order to produce reliable material by design, particularly for 3D printing applications. There is a need for further research in this area, as it is important to understand the properties of the PLA-based nanocomposite materials and their products depending on the type of processing. In spite of the critical importance, the role of graphene-carbon nanotube additives in the polymer undergoing different processing stages is not well studied with respect to their properties. Therefore, in the present study, we investigate the electrical and tensile properties of PLA nanocomposites with graphene-carbon nanotube fillers influenced by three important processing stages: filament extrusion, 3D printing, and hot-pressing. We relate the properties to the dispersion structure, morphology, and crystallinity of nanocomposites filled with 6 wt.% GNPs, multiwall carbon nanotubes (MWCNTs) and their combinations. The aim of this study is to investigate the structure-property relations of PLA nanocomposites governed by the processing conditions.

## 2. Materials and Methods

In this study, the PLA Ingeo™ Biopolymer PLA-3D850 (Nature Works, Minnetonka, MN, USA), which is specifically developed for manufacturing 3D printer monofilament, is adopted as host thermoplastic polymer. It shows remarkable 3D printing characteristics such as good adhesion to platform plates, low odor during the printing process, as well as less warping or curling, which are favorable elements for obtaining precise detail. Regarding some of its technical specifications, it is worth to note a relatively fast crystallization rate, a melt flow rate (MFR) 7–9 g/10 min (210 °C, 2.16 Kg, according to D1238 American Society for Testing and Materials (ASTM) Method), a peak melt temperature ranging in the temperature interval of 165–180 °C and a glass transition temperature of 55–60 °C. About the nanofillers, graphene nanoplates (TNGNP, from Times Nano, Chengdu, China) and multiwall carbon nanotubes (NC7000™, provided from Nanocyl, Sambreville, Belgium) are used. The nanofillers trademarks are selected based on their good characteristics and low price. Details on the basic properties of the nanofillers are summarized in Table 1.

### 2.1. Preparation of Nanocomposites and Test Samples

The nanocomposite compounds were fabricated by melt extrusion technique. The PLA pellets were ground and mixed with nanofillers in a twin screw extruder (COLLIN Teach-Line ZK25T, Maitenbeth, Germany), at temperatures 170–180 °C and screw speed 40 rpm. Mono-filler nanocomposites (GNP/PLA and MWCNT/PLA) with 6 wt.% filler contents were produced, as a masterbatch. Bi-filler composites of 6 wt.% total filler content were prepared by mixing the two mono-filler masterbatches in appropriate proportion in a second extrusion run. All compounds used in this study are processed by two extrusion runs, and they are listed in Table 2. For estimation of the electrical percolation threshold, composite formulations with filer content ranging from 1.5 to 9 wt.% are tested, which were produced in our previous study [3]. Three types of test samples are prepared for characterization: filaments, 3D printed samples, and hot-pressed specimens. The FDM filament of 1.75 mm diameter is fabricated from the nanocomposite pellets by single screw extruder (Friend Machinery Co., Zhangjiagang, China) at 10 rpm within temperature range 170–180 °C followed by quenching in the water bath at 60 °C. The 3D printed test samples are fabricated from the upper filament by FDM technique with layer-to-layer deposition at temperature 200 °C, printing speed 1000 mm/min, and platform temperature of 65 °C using German RepRap 3D printer *X*-400 Pro (German RepRap GmbH, Feldkirchen, Germany). The 3D printed samples are processed with rhomboidal printed structure. The hot-pressed samples are prepared from the composite pellets by pressing at a temperature of 180 °C and pressure of 1 bar. The sample size and shape are chosen accordingly to the test method.

### 2.2. Experimental Methods

#### 2.2.1. Field Transmission Electron Microscopy (TEM)

Bright-field transmission electron microscopy (TEM) analysis is performed on an FEI TECNAI G12 Spirit-Twin (LaB6 source, FEI Company, Hillsboro, OR, USA) equipped with an FEI Eagle-4k CCD camera, operating with an acceleration voltage of 120 kV. Before the analysis, sections of the filament samples are realized at room temperature on a Leica EM UC6/FC6 ultramicrotome and placed on 400 mesh copper grids.

#### 2.2.2. Differential Scanning Calorimetry (DSC) Analysis

Differential scanning calorimeter DSC-Q20 (TA Instruments, New Castle, PA, USA) is used to monitor the heat effects associated with phase transitions of the polymer as a function of temperature. Filaments, 3D printed strips and pressed films are tested. The 10 mg sample in a covered aluminum pan underwent a heating/cooling/heating program in the temperature range 40–200 °C. The heating and the cooling rates are 10 °C/min. From the DSC thermograms the glass transition temperature (*T*_g_), cold crystallization temperature (*T_cc_*), melting temperature (*T_m_*), melt crystallization temperature (*T_c_*), exothermic cold crystallization enthalpy (Δ*H_cc_*), endothermic melting enthalpy (Δ*H_m_*) and degree of crystallinity (*χ_c_*) are evaluated. Taking into account that the PLA undergoes cold crystallization during heating, the degree of crystallinity of the PLA-based samples is calculated by the following equation:

(1) $$\%\;\mathrm{crystallinity}\;\left(\chi_{c}\right)=\left(\frac{\Delta H_{m}-\Delta H_{cc}}{w\Delta H_{m}^{0}}\right)\times 100\;\left(\%\right)$$

where: Δ*H_m_* is the fusion/melting enthalpy (J/g), $\Delta H_{cc}$ cold crystallization enthalpy (J/g) and $\Delta H_{m}^{0}$ (93 J/g) is the melting enthalpy when the crystallinity of PLA is 100% [15]. Enthalpy values for the nanocomposites are normalized on the actual amount of polymer (*w*) involved in the thermal transition, as the filler is not involved in melting/crystallization processes. The presence of the crystalline phase transition peak (*T*_*cc*1_) is taken in view during the determination of the fusion enthalpy. This approximate Equation (1) can provide information on the crystallinity of the sample removing the effects due to cold crystallization and filler content [16].

#### 2.2.3. Electrical Measurements

**Figure 1:**
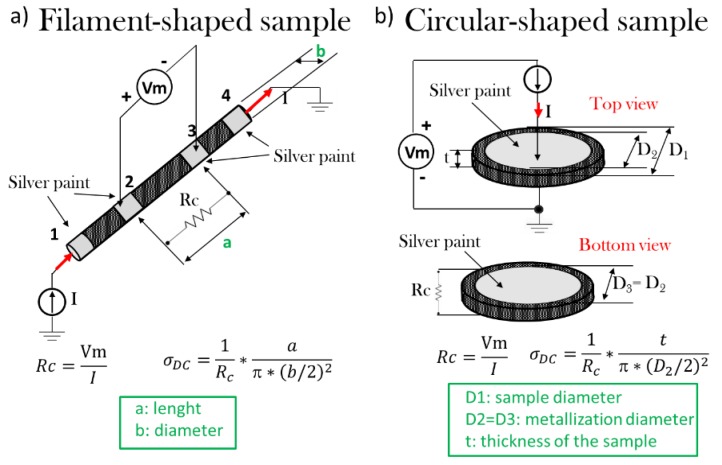
Set-up for the measurement of the direct current (DC) conductivity in (**a**) filament-shaped sample and (**b**) circular-shaped specimens in (**a**,**b**), respectively.

The direct current (DC) electrical properties are evaluated in terms of electrical percolation threshold (EPT) and electrical conductivity using an electrometer 6517B (Keithley Instruments, Cleveland, OH, USA) configured in the dual function of voltage generator (max ± 1000 V) and ammeter (current measurements from 10 aA). Figure 1 reports the schematic of the adopted set-up for the DC conductivity measurements of the filament (Figure 1a) and circular-shaped specimens prepared by 3D printing and hot-pressing (Figure 1b), respectively.

As it concerns the DC characteristics of the filament-shaped specimens characterized by a diameter *b* = 1.75 mm, a 4-probe method is applied to the four gray regions, numbered from 1 to 4, representing the metalized electrical contacts obtained with a silver paint (Alpha Silver Coated Copper Compound Screening, RS 186–3600 with volume resistivity 0.001 Ω·cm, when fully hardened). Such contacts are evenly spaced at a distance of 10 mm A suitable voltage applied between the electrodes 1 and 4, induces a current I, which in turn determines a voltage drop *V_m_* detected between the contacts 2–3. Once these electrical quantities have been measured, leveraging Ohm’s law is possible to calculate the value of the resistance *R_c_* as well as the electrical conductivity *σ_DC_* in accordance with the following analytical equations:

(2) $$R_{c}=\frac{V_{m}}{I};\;\sigma_{DC}=\frac{1}{R_{c}}\times \frac{a}{\pi \times {\left(b/2\right)}^{2}}$$

Regarding the measurements carried out on circular-shaped samples having a diameter (i.e., *D*_1_) of about 50 mm, a 2-probe method is used to determine the DC bulk conductivity of the material (i.e., *σ_DC_*) through a thickness of about 1 mm (i.e., *t*). Also, in this case, it is adopted a metalization (circular musk with a diameter *D*_2_ = *D*_3_ of 22 mm) with silver paint in order to ensure an Ohmic contact between the sample and the measuring electrodes, and to reduce the eventual surface roughness. Once the voltage and current values (i.e., *V_m_* and *I*, respectively) are known, it is possible to calculate the resistance of the material (i.e., *R_c_*), and therefore, its electrical conductivity using the following relationships:

(3) $$R_{c}=\frac{V_{m}}{I};\;\sigma_{DC}=\frac{1}{R_{c}}\times \frac{t}{\pi \times {\left(D_{2}/2\right)}^{2}}$$

Although five samples for each composition are prepared and then experimentally characterized, the electrical data reported as results refer to the average values. In any case, before the electrical measurements, all samples are thermally pre-treated at 40 °C for 24 h in order to avoid humidity effects and to favor the evaporation of any residual solvents or chemical agents.

#### 2.2.4. Tensile Test

Tensile mechanical measurements are carried out on Universal Mechanical Testing Machine (UMT-2, Bruker, Campbell, CA, USA). Tests are performed at room temperature by using 1 kN force sensor, with a tensile speed of 1 mm/min. The filament-shaped samples have 50 mm length and 1.75 mm in diameter. The 3D printed, and the hot-pressed samples are standard dog-bone shaped specimens with 50 mm in length and 3 × 1 mm cross-section of the working zone. The mean values of tensile characteristics and the standard deviation are determined by testing of 7–10 samples of each composition. During the experiment, the working section of the sample under tension was set of 1 cm. The tensile mechanical characteristics determined from the test are namely the Young’s modulus, yield strength, ultimate strength, elongation at ultimate strength, and toughness.

## 3. Results and Discussion

### 3.1. Morphological Investigation

**Figure 2:**
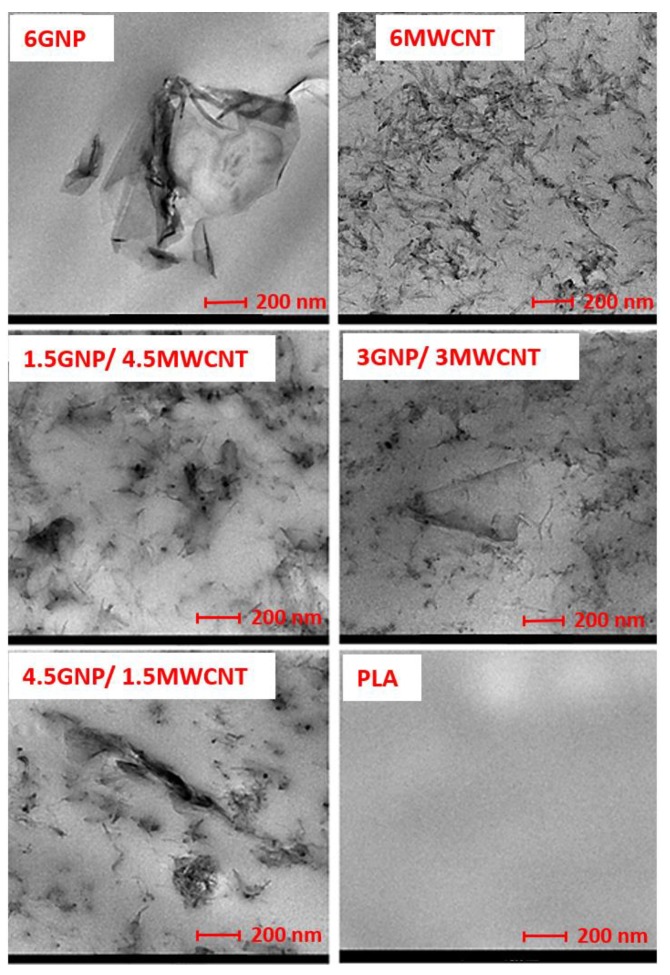
TEM images of the mono-filler and bi-filler composites at 6 wt.% filler contents, and neat poly(lactic) acid (PLA), compared at the same magnification.

Morphological analysis of the filament is performed by transmission electron microscopy (TEM), in order to state the degree of dispersion of the fillers in the PLA matrix as a function of the filler type and composition. Samples are thin slides cut from the cross-section of the filament. Figure 2 (first line) shows the TEM images of the 6 wt.% mono-filler GNP/PLA and MWCNT/PLA composite filaments, while the second and third lines present the bi-filler composites of ratio 1.5GNP/4.5MWCNT, 3GNP/3MWCNT and 4.5GNP/1.5 MWCNT, respectively. All images are compared at the same magnification.

As seen from Figure 2, the state of dispersion and filler distribution in the mono-filler composites based on poly(lactic) acid/graphene nanoplates (GNP/PLA) and poly(lactic) acid/multiwall carbon nanotubes (MWCNT/PLA) at 6 wt.% filler contents depends strongly on the type of filler. The 6GNP/PLA filament (first line, left) demonstrate an aggregated structure with broken continuity where graphene particles vary in size from 200 nm to ~2 µm. In contrast, the 6MWCNT/PLA (right) demonstrates a segregated network structure where carbon nanotubes form continuous pathways in the polymer matrix. In the bi-filler composites (Figure 2, second, and third lines), a synergic effect of filler combination on the state of dispersion is observed, which depends on the filler ratios. Thus, in the bi-filler 1.5GNP/4.5MWCNTs filament, a segregated network is formed consisting of joint agglomerates forming continuous pathways in the polymer matrix. While, in the bi-filler 3GNP/3MWCNTs system, a homogeneous network of randomly distributed and well-dispersed MWCNTs and thin GNPs is organized. However, at filler ratio 4.5GNP/1.5MWCNT, a mixed segregated-aggregated structure with partly broken continuity is formed where the MWCNTs are concentrated on the surfaces of the large graphene platelets in the PLA matrix.

The different structural types and morphology formed in the mono-filler and the bi-filler composite filaments, such as the segregated network, homogeneous network, and aggregated structure will be discussed herewith in the next sections, in context to their influence on the electrical conductivity and tensile mechanical properties of nanocomposites.

### 3.2. DSC Characteristics

**Figure 3:**
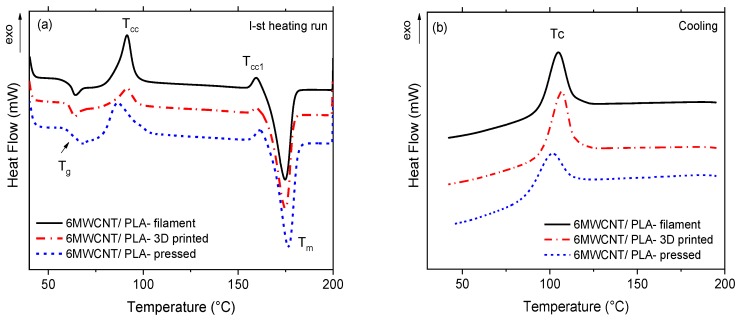
Differential scanning calorimetry (DSC) thermograms for 6 wt.% poly(lactic) acid/multiwall carbon nanotubes (MWCNT/PLA) nanocomposite: (**a**) first heating run and (**b**) subsequent cooling, comparing the filament, the 3D printed, and the hot-pressed samples.

**Figure 4:**
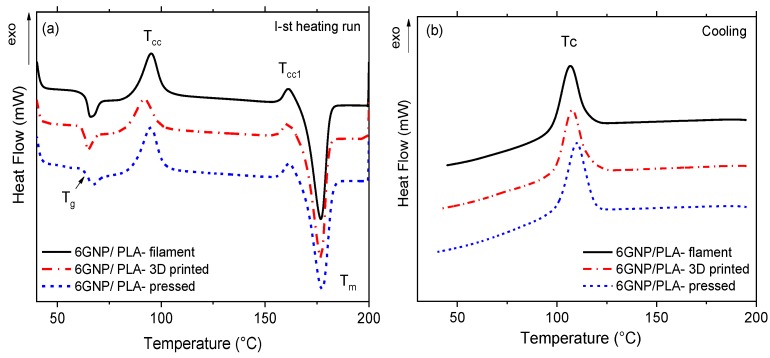
DSC thermograms for 6 wt.% GNP/PLA nanocomposite: (**a**) first heating run and (**b**) subsequent cooling, comparing the filament, the 3D printed, and the hot-pressed samples.

The influence of different thermo-mechanical processing conditions upon the thermal behavior of the filament, the 3D printed strips, and the hot-pressed samples is studied by DSC analysis. The “as received” samples are tested using the first DSC heating run, followed by subsequent cooling of the melt. The results are shown in Figure 3 and Figure 4, as well as the numerical values of thermal characteristics, are summarized in Table 3.

In Figure 3, the DSC thermograms of the 6 wt.% MWCNT/PLA composite are presented: (a) first heating run and (b) melt cooling, where the filament, the 3D printed and the hot-pressed samples are compared. Figure 4 shows the respective DSC thermograms for the 6 wt.% GNP/PLA composite.

In general, the thermograms demonstrate several typical thermal events during heating, such as glass transition (*T_g_*), cold crystallization (*T_cc_*), crystalline phase transition peak (*T*_*cc*1_) and melting (*T_m_*), as well as the melt crystallization peak (*T_c_*) during cooling. When considering the effect of the processing stage, Table 3 shows that the composite filament and the 3D printed strips demonstrate similar characteristic temperatures, which are insufficiently influenced by the type of filler. Therefore, we may conclude that both GNPs and MWCNTs have similar effects on the PLA chain relaxation around *T_g_*, the cold crystallization process, the melting of crystals and melt crystallization during cooling, which are insufficiently affected by both extrusion processing stages, the filament production and the 3D printing (FDM). In contrast, only glass transition and a small melting peak were evident for the neat PLA filament during heating. The absence of a melt crystallization peak for the neat PLA filament during cooling indicates that the homopolymer is difficult to be crystallized from the glassy state; thus, it is almost amorphous. The melting temperature (*T_m_*) of the neat PLA filaments appeared at 25 °C lower temperature compared to the composite filament, which is associated with the melting of a large portion of crystals in composites, which are formed due to the nucleation effect of GNPs and MWCNTs.

However, for the hot-pressing stage, a small difference was found for hot-pressed MWCNT/PLA sample, which shows 5–7 °C lower values of the cold crystallization (*T_cc_*) and the melt crystallization (*T_c_*) peaks compared to that of the filament, and the 3D printed one. This is indicative of the nucleation effect of MWCNTs, which facilitate cold and melt crystallization of PLA during the hot-pressing stage, due probably to the high pressure and slow cooling. However, such effect is not observed for the GNP/PLA composite, and its characteristic temperatures are similar for the three processing stages.

Importantly, Table 3 shows that the 3D printed samples show much higher % crystallinity (41.7% for 6MWCNT and 34.3% for 6GNP) compared to that for the filament (30% and 26%) and for the hot-pressed samples (28%), respectively. This is produced by lower cold crystallization enthalpy (Δ*H_cc_*) and higher melting enthalpy (Δ*H_m_*) of the 3D printed strips compared to that of filament and hot-pressed one. Thus, for the 3D printing stage, the nucleation effect of MWCNTs and GNPs is stronger during the melt crystallization and weaker during the cold crystallization of PLA. A small difference is observed in the cold crystallization enthalpy, where the nucleation effect of GNPs is slightly stronger than that of MWCNTs. The most likely reason for such variations in the nucleation and percentage crystallinity is the difference in heating and cooling rates in the three processing stages, which govern the crystallization process of PLA. Obviously, the layer-to-layer deposition during 3D printing FDM provides a lower cooling rate, compared to the filament extrusion and hot-pressing; thus, giving more time for nucleation and resulting in higher percentage of crystallinity.

In general, the results show that the 3D printing process produced a higher degree of crystallinity of PLA than the filament extrusion and the hot pressing. For the three processing stages, the nucleation effect of GNPs is slightly weaker compared to MWCNTs, this confirming Li at al. [11], reporting that such difference is due to the multiple orientations of PLA lamellae on the large two-dimensional flat surface of GNPs, which suppressed the crystal growth. How the percentage of crystallinity of the 3D printed samples compared to the filament and hot-pressed samples influence the properties of nanocomposite materials will be discussed herewith in the next sections.

### 3.3. DC Electrical Properties

**Figure 5:**
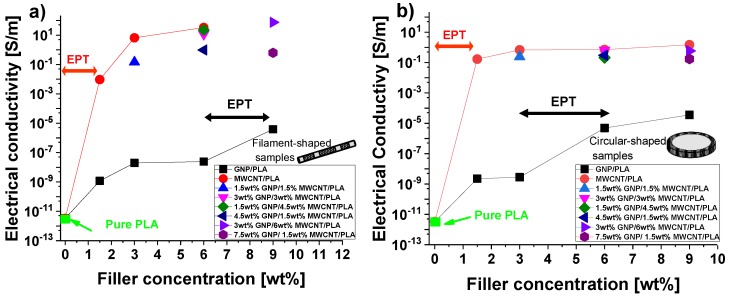
Electrical conductivity as a function of the filler concentration (wt.%) for the different formulations of (**a**) filament and (**b**) hot-pressed samples.

Figure 5 shows the DC electrical conductivity for filaments of PLA reinforced with different amounts of multiwall carbon nanotubes (MWCNT/PLA), graphene nanoplates (GNP/PLA) or a combinations thereof (GNP/MWCNT/PLA) for filament-shaped and circular samples (hot-pressing made), in Figure 5a,b, respectively. It should be noted, in particular, the absence of the filament at 9 wt.% MWCNTs that could not be produced due to the extrusion issues at this high concentration, when using this type of filler exclusively. The starting electrical conductivity of pure PLA is a few pS/m, as expected for insulating material.

Consistent with percolation theory, as soon as the filler concentration increases, the electrical resistivity of material reduces, and therefore, its electrical conductivity increases to as high as several orders of magnitude compared to the unfilled PLA. This phenomenon indicates that electrically conductive paths between strictly close nanoparticles are established within the material; thus, inverting its behavior from insulating to conductive one since the electrons can flow due to tunneling effect. About the filament-shaped specimens, at the concentration of 6% by weight of filler, although PLA reinforced with GNPs still showing a low value for the electrical conductivity (~2.4 × 10^−8^ S/m), an interesting value of about 33 S/m was measured for MWCNTs-based filaments. Remarkable and comparable values are also measured in filaments containing both fillers with an evident improvement of the electrical conductivity with increasing concentration of nanotubes (1.5, 3, 4.5 wt.% MWCNTs) adopted in the prepared formulations. The combined use of both carbon-based fillers, due to their synergic effect on the dispersion (as discussed by morphological analysis in Section 3.1), favors the formation of the electrical network allowing to obtain an electrical conductivity of about 74 S/m at the highest investigated filler concentration (9 wt.% of total charge with 1:3 and 2:3 of GNP/MWCNT, respectively). Some consideration deserves the substantial difference between the two electrical percolation thresholds (EPT), i.e., the minimum amount of filler to obtain the inversion of the electrical behavior of the material (discussed above), found for filaments made with nanotubes rather than graphene nanoplates. For the MWCNTs, it has already been achieved with 1.5 wt.%, whereas for the GNPs, the EPT falls in the wider range 6–9 wt.%. Such discrepancy may be justified by considering that the percolation threshold in polymer nanocomposites based on electrically percolating networks is affected by strongly influencing parameters such as the manufacturing process, the nature of matrix and its interaction with the filler, the aspect ratio, the functionalization, the dispersion state and the tendency to agglomerate of this latter and so on [17,18,19,20].

About the disc-shaped samples, for MWCNTs-based composites, the EPT is confirmed with 1.5 wt.% of filler whereas for those filled with GNPs, the EPT is observed in the higher range 3–6 wt.%. The considerations made above for filament-shaped filaments on the differences in the EPT correlated to the adopted fillers remain still valid for the same reasons for such samples. Except for PLA reinforced with GNPs, a slight worsening (almost an order of magnitude) of the electrical conductivity is observed. In fact, at the highest investigated filler concentration (9 wt.%), a maximum value of 1.49 S/m is measured for the electrical conductivity of carbon nanotube-based composites.

In any case and regardless the type of dispersed filler and the fabrication process, the electrical conductivity (i.e., $\sigma_{DC}$) above the percolation region follows with the increasing of the loading (i.e., ϕ) a characteristic trend described by a power law:

(4) $$\sigma_{DC}=\sigma_{0}{\left(\phi -EPT\right)}^{t}$$

where $\sigma_{0}$ is the intrinsic conductivity of the filler, and *t* is a critical exponent depending on the dimensionality of the percolating structure [21].

**Figure 6:**
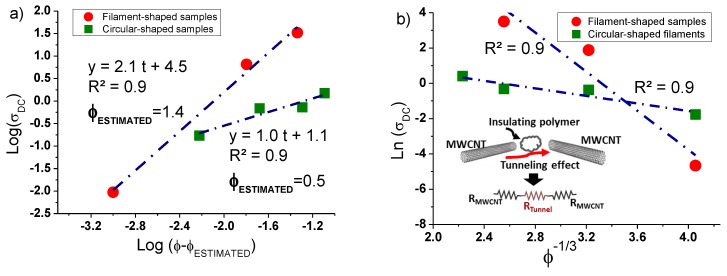
For multiwall carbon nanotubes (MWCNTs)-based composites: (**a**) Log-log plot of the electrical conductivity as a function of (ϕ-ϕ_estimated_); (**b**) Plot of the natural logarithm of DC conductivity for the sample above the electrical percolation threshold (EPT) vs. ϕ^−1/3^. In both cases, the dashed lines fit the DC data (red and green markers).

In particular, the characteristic parameters of the percolation law can be estimated by examining the log-log plots of the experimental conductivity data as a function of the filler loading, as reported in Figure 6a. The case of MWCNT/PLA is taken into consideration since it is necessary to have a minimum number of experimental data above the EPT (at least three). Consistently with the results reported in the previous Figure 5. The analysis is conducted on filament-shaped and circular samples (hot-pressing made).

More specifically, the critical exponent *t* coincides with the slope of the linear fit of the data, whereas the value of the estimated electrical percolation threshold (i.e., ϕ_estimated_) is assumed as an adjustable parameter to maximize the regression coefficient (i.e., R^2^ → 1) for the interpolating curve. The value of the exponent *t* (i.e., *t* = 2.1) agrees with predictive literature studies [21], whereas the estimated EPT of 1.4 is very close to that experimentally found. For hot-pressed made composites, the value of this exponent reduces to 1 since the mechanical load forces the arrangement of the filler in a two-dimensional space rather than 3D [21]. The estimated percolation threshold is around 0.5 wt.%.

An approach adopted in literature [22,23,24] aimed to confirm that the electron tunneling is the principal electrical transport mechanism in nanofilled structures is based on the verification of a linear relation (see Figure 6b) between the electrical conductivity (in natural logarithmic scale) and ϕ^−1/3^, valid for concentrations (ϕ) above the EPT, i.e., *ln* (*σ_DC_*) ∝ ϕ^−1/3^. Also, for such analysis, only the case of MWCNT/PLA can be taken into account for the same reasons already explained. The dashed line is a fitting curve (R^2^ very close to 1) of the experimental data (red and green markers). Given the occurrence of the linearity condition, it is plausible to assume that the electrical conduction in the analyzed composites is mainly due to the quantum tunneling effect. This last can be quantified by means of an electrical resistance which is strongly affected by several parameters in agreement with the following equation adopted for its quantification:

(5) $$R_{\mathrm{tunnel}}=\frac{h^{2}d}{A_{\mathrm{tunnling}}e^{2}\sqrt{2m\lambda }}exp^{\left(\frac{4\pi d}{h}\sqrt{2m\lambda }\right)}$$

where *h* is the Plank’s constant, *d* is the interparticle distance, *e* and *m* are, respectively, the electron charge and the electron mass, *λ* represents the height of barrier, and *A*_tunneling_ is the area involved in the electron tunneling. In particular, interparticle distances and height of barrier play a key role in such effect, and above all, they are conditioned not only by the intrinsic features of the filler but also by the dispersion technique and manufacturing process as well as the mutual interaction between host matrix/filler and interfacial effects. For this reason, many research efforts are needed for the development of a controllable production process in order to obtain customized and reproducible materials.

**Figure 7:**
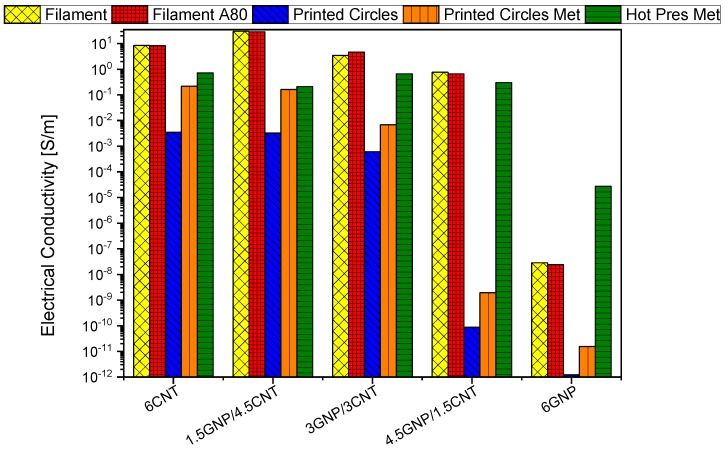
Comparison in terms of DC conductivity of the filament-shaped, the 3D printed and hot-pressed circular samples at 6 wt.% filler concentration. Effects due to filament annealing and metallization of the test samples are also investigated.

Figure 7 shows a comparison in terms of electrical conductivity of the filament, 3D printed strips, and pressed samples at 6 wt.% filler content in order to verify the influence of the three processing stages, and the annealing on the final electrical performances. This concentration was chosen because it is the highest achieved in the MWCNT/PLA filament composites as well as a concentration which, although limited, allows to obtain interesting conductivity values comparable to those exhibited by composites filled with higher nanoparticles loading.

Importantly, the filament-shaped samples show the highest value of conductivity, followed by the hot-pressed and then the 3D printed samples. Let us consider the effect on the conductivity of the three structural types formed in the mono-filler and the bi-filler composite filaments (reported in Section 3.1). Figure 7 (yellow bars) showed that the segregated network of 6% MWCNT and 1.5 GNP/4.5MWCNT produced the highest electrical conductivity (~10 and ~30 S/m, respectively), while the homogeneous network of 3GNP/3MWCNT resulted in lower conductivity (~4 S/m). Much lower conductivity (~0.8 S/m) is observed for the mixed segregated-aggregated structure of 4.5GNP/1.5 MWCNT due obviously to the locally broken continuity. Finally, the aggregated structure of 6% GNP shows conductivity of ~10^−8^ S/m, which is below the electrical percolation.

On filament-shaped samples, the effect of a solid-state annealing performed at 80 °C for 4 h (i.e., above the glass transition temperature, *T_g_*~65 °C but below the cold crystallization temperature, *T_cc_*~100 °C) was investigated. Solid annealing filaments (red columns) show electrical conductivity values comparable to those of non-heat-treated filaments (yellow columns). Most likely, there are no appreciable variations due to poor mobility of the polymer chains slowed by matrix-filler interfacial adhesion. As a consequence, the morphology of the percolation network has remained substantially unchanged, and therefore, the electrical conductivity has not been influenced as also observed by Kotsilkova et al. [14] whereas a moderate conductivity enhancement, after annealing process at higher temperature, due to a better-interconnected network between conductive particles favored by viscoelastic relaxation of the polymer are discussed in [13,25,26]. In fact, a multiplicity of time-temperature conditions were experimented for annealing of PLA, and different behaviors are consequently observed since it is, in any case, an efficient treatment to increase the modulus and tensile strength of such material [14,27,28,29].

By using a 3D printer and the produced filaments as starting materials, circular-shaped samples were obtained. The electrical conductivity of these resulting materials is definitely lower (blue columns) than that of the filaments. The reason could be of dual nature. Certainly, on the one side, as reported in [30], there are a lot of main factors influencing the quality of the final printed objects such as the parameters setting concerning the extrusion temperature and its speed, the platform temperature, the infill percentage, the printing direction and more generally, the need to adapt the physical-chemical characteristics of the filaments to the specific features of the printer. On the other hand, in the filaments, as they were produced through an extruder, there could be a sort of preferential direction for the nanoparticles which favor the percolation network; thus, improving their electrical conductivity, as pointed above. All these aspects may be associated with anisotropy of electrical properties that will be investigated and discussed in a future paper.

The use of metallization for both samples surfaces with silver paste, as described in the section “Electrical measurements”, is particularly useful for printed objects, as it is well known that one of the main drawbacks of this technology is the excessive roughness of the resulting superficial parts. The silver coating, smoothing the surfaces, reduces this inconvenient, as well as the contact resistance with the measuring electrodes and consequently a significant improvement in the electrical conductivity (orange columns), is observed with respect to the non-metalized samples.

With the hot-pressing manufacturing approach, it is possible to achieve good performances, especially in terms of electrical conductivity, since the mechanical action of the process leads to a reduction of interparticle distances (term *d* of the above Equation (5)). Consequently, the tunneling resistances in the percolated network are reduced, and the overall electrical conductivity of the resulting materials increases.

### 3.4. Tensile Properties

**Figure 8:**
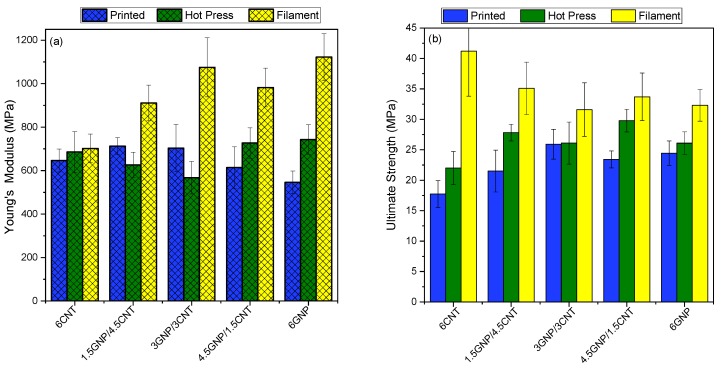
Tensile Young’s modulus (**a**) and ultimate strength (**b**) of 6 wt.% mono-filler and bi-filler composites by varying the filler combinations. The mean values and standard deviation are presented.

Series of tests are performed to evaluate the tensile mechanical characteristics of the filaments, the 3D printed, and the hot-pressed samples, composed of mono-filler and bi-filler composites at 6 wt.% filler content. The effects of the three processing stages on mechanical behavior, combined with the reinforcement effect of graphene-carbon nanotube additives are evaluated and related to the structural features, as the filler distribution and crystallinity (discussed in Section 3.1 and Section 3.2). The Young’s modulus, yield strength, tensile strength, elongation, and toughness are determined, and the results are summarized in Figure 8 and Table 4. The 6 wt.% filler content was chosen based on our previous study [3] showing the maximum improvement of tensile characteristics of the filament within the range of 1.5 to 9 wt.% filler content.

In general, the filaments (yellow bars in Figure 8) demonstrate of 50–150% enhanced tensile characteristics compared to the 3D printed (blue bars) and the hot-pressed (green bars) samples. This effect could be associated with anisotropy of tensile properties due to the preferential orientation of nanoparticles along the length of the filament during extrusion. Obviously, the rhomboidal structure of 3D printed samples cannot provide such anisotropy.

The high standard deviations of the mean values of tensile characteristics confirm the local inhomogeneity that may appear due to the various nanoparticle orientation, from random to aligned, along the filament length. As seen for the filament samples, the 6% GNP additives produced ~60% enhancement of Young’s modulus compared to the 6% MWCNT. For the bi-filler filaments, 4.5GNP/1.4MWCNT and 3GNP/3MWCNT, Young’s modulus is increased by 40–50%, compared to the 6% MWCNT; therefore, no synergic effect of the two fillers on the tensile modulus is observed.

Therefore, the highest reinforcement of Young’s modulus could be achieved by the aggregated structure, followed by the homogeneous network structure of the nanocomposites.

Moreover, the high specific surface area of GNPs may carry high levels of transferring stress across the filler-polymer interface [31]. While, the highest values of the yield strength, ultimate strength, elongation, and toughness, as well as the lower Young’s modulus of the 6 wt.% MWCNT, followed by the 1.5GNP/4.5MWCNT (Table 4), may be associated with the segregated network structure of the nanocomposites, as well as the good dispersion of carbon nanotubes allowing to express the unique mechanical strength of the individual MWCNT [32].

In contrast, the samples produced by 3D printing (FDM) and hot pressing show similar tensile characteristics, much lower than that of the filaments. Moreover, the filler types and combinations, as well as the structural organization, have moderate effects on tensile properties of 3D printed and hot-pressed samples.

The 3D printed bi-filler composites demonstrate 10–30% higher Young’s modulus and ultimate strength compared to the mono-filler systems, which could be associated with synergism.

The almost 20–50% higher crystallinity of the 3D printed samples have insufficient reinforcement effect on the tensile properties compared to that of the hot-pressed samples.

## 4. Conclusions

The effects of three processing stages, i.e., filament extrusion, 3D printing (FDM), and hot-pressing on electrical conductivity and tensile mechanical properties of poly(lactic) acid composites filled with 6 wt.% MWCNTs, GNPs, and combined fillers are studied. The processing stages influence significantly the properties of the composites studied herewith. In general, the filaments show a few decades higher electrical conductivity and 50–150% higher tensile mechanical characteristics, compared to the hot-pressed and the 3D printed samples, due probably to the preferential orientation of nanoparticles during the filament extrusion. However, the 3D printed and hot-pressed samples show relatively slight differences in electrical conductivity and tensile characteristics whereas the highest crystallinity of the 3D printed samples compared to others resulted in insufficient mechanical reinforcement.

The effects of filler distribution in the matrix polymer are considered in terms of the electrical and tensile properties of the filaments. The segregated network structure, most pronounced in the 6MWCNT and the 1.5GNP/4.5MWCNT filaments, resulted in the highest values of electrical conductivity (10–30 S/m), as well as the highest yield strength, ultimate strength, elongation, and toughness, but the quite low Young’s modulus. Instead, the homogeneous network structure of randomly distributed and well-dispersed GNPs and MWCNTs, presented in the 3GNP/3MWCNT filament, as well as the mixed segregated-aggregated structure of the 4.5GNP/1.5MWCNT filament, resulted in lower values of conductivity (~4 S/m and 0.8 S/m, respectively) and 40–50% higher Young’s modulus than that of 6MWCNT composite having a segregated network structure. In contrast, the highest reinforcement of Young’s modulus (60%) and very low conductivity (~10^−8^ S/m), below the percolation threshold, is found for the 6% GNP filaments having an aggregated structure.

Since different printing factors, such as the bed and extruder temperature, the printing orientation, the infill percentage, the cooling fan speed and so on, may affect the overall mechanical and electrical properties of 3D printed parts giving rise to anisotropic materials [33,34,35], a future paper will be dedicated exclusively to such investigation.

## Figures and Tables

**Table 1:** Features of the adopted graphene nanoplates (GNPs) and multiwall carbon nanotubes (MWCNTs).

Property	GNP Filler	MWCNT Filler
Commercial Code	TNGNP	NC7000™
Purity (wt.%)	99.5	90
Thickness (nm)	4–20	-
Average size (µm)	5–10	-
External diameter, (nm)	-	9.5
Average Length (µm)	-	1.5
Surface area (m^2^/g)	-	250–300
Volume resistivity (Ω cm)	4 × 10^−4^	10^−4^
Aspect ratio	~500	~150

**Table 2:** List of mono-filler and bi-filler nanocomposite compounds produced for this study.

Sample	PLA Content, wt.%	GNP Content, wt.%	MWCNT Content, wt.%
PLA	100	-	-
6GNP	94	6	-
6MWCNT	94	-	6
3GNP/3MWCNT	94	3	3
1.5GNP/4.5MWCNT	94	1.5	4.5
4.5GNP/1.5MWCNT	94	4.5	1.5

**Table 3:** Thermal characteristics determined by DSC first heating run and subsequent cooling.

Composites		*T_g_* [°C]	*T_cc_* [°C]	*T_cc_*_1_ [°C]	*T_m_* [°C]	*T_c_* [°C]	Δ*H_cc_* [J/g]	Δ*H_m_* [J/g]	*χ_c_* [%]
**PLA**	Filament	65.0	-	-	149.6	-	-	1.47	1.6
**6 CNT**	Filament	62.5	91.5	159.5	174.8	104.8	18.5	44.6	29.8
	3D print	62.5	91.6	159.9	174.9	106.7	10.8	47.2	41.7
	Pressed	65.7	86.6	161.3	176.5	101.4	19.9	44.8	28.4
**6 GNP**	Filament	64.8	95.2	161.3	176.9	106.7	21.5	44.16	26.0
	3D print	63.8	92.0	161.0	176.8	107.6	18.4	48.34	34.3
	Pressed	65.4	94.9	161.6	177.3	109.9	21.9	46.34	28.0

**Table 4:** Tensile characteristics of filaments, 3D printed, and pressed samples.

Filament Samples	Young’s Modulus, MPa	Ultimate Strength, MPa	Yield Strength, MPa	Elongation at Ultimate Strength, %	Toughness J/mm^3^
6MWCNT	702 ± 66	41.2 ± 7.4	7.8 ± 1.0	9.4 ± 0.5	2.0 ± 0.4
1.5GNP/4.5MWCNT	911 ± 82	35.1 ± 4.3	6.4 ± 0.3	6.2 ± 0.6	1.1 ± 0.2
3GNP/3MWCNT	1075 ± 136	31.6 ± 4.4	4.3 ± 0.6	5.1 ± 0.3	0.8 ± 0.2
4.5GNP/1.5MWCNT	982 ± 89	33.7 ± 3.9	6.1 ± 0.6	5.6 ± 0.6	0.9 ± 0.2
6GNP	1122 ± 108	32.3 ± 2.6	6.0 ± 0.6	4.9 ± 1.5	0.8 ± 0.2
**3D printed Samples**					
6MWCNT	646 ± 53	17.7 ± 2.2	4.6 ± 0.6	6.0 ± 0.3	0.7 ± 0.1
1.5GNP/4.5MWCNT	713 ± 39	21.5 ± 3.4	2.8 ± 0.4	4.0 ± 0.5	0.4 ± 0.1
3GNP/3MWCNT	703 ± 109	25.9 ± 2.5	5.1 ± 0.8	5.6 ± 0.7	0.7 ± 0.2
4.5GNP/1.5MWCNT	614 ± 96	23.4 ± 1.4	4.0 ± 0.8	4.8 ± 0.6	0.5 ± 0.1
6GNP	546 ± 93	24.4 ± 2.0	4.4 ± 1.4	6.0 ± 0.2	0.7 ± 0.1
**Hot-pressed Sample**					
6MWCNT	686 ± 94	22.0 ± 2.7	4.3 ± 0.2	5.1 ± 0.4	0.7 ± 0.1
1.5GNP/4.5MWCNT	627 ± 58	27.8 ± 1.4	2.0 ± 0.9	3.7 ± 0.4	0.4 ± 0.1
3GNP/3MWCNT	568 ± 75	26.1 ± 3.5	1.8 ± 0.5	6.2 ± 0.8	0.8 ± 0.2
4.5GNP/1.5MWCNT	727 ± 70	29.8 ± 1.9	2.1 ± 0.3	6.2 ± 0.6	0.9 ± 0.1
6GNP	743 ± 68	26.1 ± 1.9	5.3 ± 0.5	6.2±0.7	0.9±0.2
